# Sustainable business model for local council’s smart city initiatives: a systematic literature review

**DOI:** 10.12688/f1000research.73373.1

**Published:** 2021-10-20

**Authors:** Ezatul Faizura Mustaffa Kamal Effendee, Magiswary Dorasamy, Abdul Aziz Bin Ahmad, Azrin Aris, Saida Harguem, Maniam Kaliannan

**Affiliations:** 1 Faculty of Management, Multimedia University, Cyberjaya, Selangor, 63100, Malaysia; 2 Telekom Malaysia Bhd, Kuala Lumpur, Wilayah Persekutuan, 60000, Malaysia; 3 Canadian University Dubai, Dubai, United Arab Emirates; 4 University of Nottingham Malaysia, Semenyih, Selangor, 43500, Malaysia

**Keywords:** smart city, local authority, business model, frugal innovation, financial sustainability, municipality, resilience

## Abstract

**Background:** Malaysia is embarking on sustainable, resilient, and prosperous living conditions initiatives. Malaysian cities are embracing the smart city aspiration through their respective local authorities. However, they face challenges regarding  funding allocation for smart city implementation. Local authorities primarily operate on a conventional business model. Based on their current business model, they are unlikely to sustain their smart city initiatives. A more financially sustainable business model is required by these local authorities to embark on smart city initiatives. This study presents a systematic review concerning the business models adopted by local authorities to implement smart cities. This paper also explores the applicability of frugal innovation towards developing a smart city business model.

**Methods:** This article undertakes a systematic review based on combination sets of eight main keywords: smart city, business model, frugal innovation, local authorities, performance, inclusivity, technology and success factor. The search strategy includes journal articles and conference proceedings from five major online databases: Emerald, ProQuest, Scopus, IEEE Xplore, ScienceDirect, and Springer Link between 2001-2021. The data is tabulated for clear expression of knowledge gaps.

**Results:** A total of 17 articles from 300 articles on smart city business models matched the search on smart city business models for local authorities . The study revealed that hardly any in-depth research providing the crucial elements for a successful smart city business model for local authorities has been conducted. No research has linked frugal innovation to smart city business models.

**Conclusions:** The study calls upon the research community to explore further, the possible linkage between frugal innovation and smart cities for local authorities.

## Introduction

In Malaysia, many goals have been adopted as compasses for smart city initiatives at the national and global level, such as the United Nation’s Sustainable Development Goals, the New Urban Agenda, the Twelfth Malaysia Plan, the National Physical Plan 3 and the National Urbanisation Policy 2. Malaysia currently has 150 local councils or ‘Pihak Berkuasa Tempatan’ (PBT), comprising 19 city councils, 37 municipal councils and 94 district councils. ^1^ Through their respective PBTs, cities around Malaysia are beginning to embrace digital city services, in line with the global movement to achieve smart and sustainable cities. However, they do not have enough funding allocated for that purpose. If they continue to operate on their conventional operations model, how can cities even hope to start embarking on the smart city aspiration when they do not have the financial means to do so? With the many challenges surrounding the financing of smart cities, practitioners and academics are exploring alternative methods. Traditional business models mainly consider how much value can contribute to the equation. However, with the increasing complexity of the smart city ecosystem, new business models must also be considered. This study contributes toward one of the 10 priority areas of the 10-10 MySTIE framework and provides insight and ways forward for smart city knowledge community.

### Smart city phenomenon

The year 2007 marked a significant milestone in modern urban living, when the number of city dwellers across the world, tipped the number of those living in rural areas. ^2^ This number is not expected to slow down anytime soon, with almost two-thirds of the world’s population expected to live in urban areas by 2050. ^2^ This mass migration to the cities has inevitably strained city resources and given rise to social issues. City authorities, developers, town planners, and all those tasked with providing a better standard of living have been forced to find better, cheaper, and more efficient solutions. Fortunately, the advancement of information and communication technology (ICT) supporting these services is quite advanced. The stakeholders can leverage these technologies to ensure a better quality of life for city inhabitants. Thus, the term ‘ smart cities’ has been coined; whereby cities embark on initiatives that leverage ICT and technology to provide a better quality of life.

One significant challenge with smart city projects is the investments needed. In a conference held in 2018, ^3^ the Iskandar Regional Development Authority of Malaysia (“IRDA”) presented the Malaysian government’s aspiration for the Iskandar Malaysia smart city. One of the items mentioned was on the matter of funding, in regards that more smart city developments need private organizations’ support to fund and sustain the cities. Traditionally, cities are funded by government, for example, through tax collections. However, with funds getting more depleted, cities pursuing smart city initiatives need to find alternative sources of income; thus, the need for private investments. ^4^ However, one significant challenge with private corporations’ involvement in smart cities is the business model, where traditional government-funded projects are predominately driven by altruistic motivation. Private corporations’ obligation is ultimate to their shareholders, where expectations are simple: a healthy bottom line.

Strategic smart city initiatives often come with non-financial benefits, for example, data. Smart cities collect a lot of data from many data points. The Selangor Smart City initiative for example, involves implementation of many digital services to facilitate city dwellers’ everyday living, including CCTV, intelligent traffic management systems, smart parking, cashless payments, air quality indicators and water quality indicators. If analysed and synthesised correctly, these data can be transformed into other meaningful information that can be useful to many parties. However, the question of how these data can be properly and effectively monetised is still in its infancy. Until these issues can be resolved, the data collected, though valuable, would be difficult to be valued and quantified for financial analysis. Investment in smart cities is fraught with challenges; returns are uncertain, business models are still in infancy, and investments take a long time to recover. ^5^ Thus, perhaps many smart cities would not have been realised if decision-makers had relied solely on financial motivations. With the many challenges surrounding the financing of smart cities, practitioners and academics are exploring alternative approaches for sustainable smart city funding. With the increasing complexity of the smart city ecosystem, new business models need to be explored; ideally where the city can financially sustain its smart services. The frugal innovation concept is about doing more with less, by reducing complexity and focusing on delivering the actual needs. ^6^ This approach has been used mainly in engineering and industrial design, but how the same concept can also be applied to smart city business models is worth investigating.

Given that backdrop, the research questions for this study are as follows:

1. How extensive is the literature coverage for smart city business models, specifically concerning local authorities?
2. For the literature that covers the above, what are the business models proposed, if any?
3. Can frugal innovation theory be applied to develop a smart city business model?
  The objectives of this research are as follows:
1. To investigate the extent to which business models for local authorities’ smart city initiatives are covered by literature.
2. To identify any business models that have been proposed for local authorities to deploy smart city initiatives successfully.
3. To investigate whether frugal innovation theory can be applied to the smart city business model.

## Methods

### Ethics

This study was conducted according to the guidelines and approved by the Research Ethical Committee of Multimedia University, Malaysia (EA1402021).

**Figure 1.**
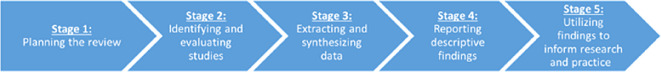
Tranfield’s five stages of systemic review.

This study was designed to present a literature review, research gap analysis, and insights on the extent to which business models for local authorities’ smart city initiatives are covered by literature. This literature review was based on the Tranfield’s five stages of systemic review ^7^ as shown in Figure 1.

### Stage one: planning the review

The main goal of this review is to ascertain the nature and form of research about business models adopted by local authorities to deploy smart city initiatives. The paper aims to offer researchers a comprehensive review of previous works related to smart city business models. The outcome is to offer the smart city community a series of research ideas to move the field forward.

### Stage two: identifying and evaluating studies: planning the review

One of the issues identified that hindered the identification of all papers that analysed business models adopted by local authorities is that other terms are also used to refer to ‘local authority’, such as local councils and municipalities. Therefore, for this study, all three terms were used and cross-referenced with other terms, including smart city, business model and frugal innovation. The study then reviewed all the available literature and excluded those that were irrelevant.

*Inclusion and exclusion criteria*

To perform the literature review and ensure only quality studies were included, it was decided to include journal/conference papers and research articles from 2001 to 2021. The keywords that were used to select the studies are further explained in the section below.

Regarding the exclusion factors, the study excluded books, book chapters and dissertations from the sampling frame to keep the studies’ quality high, with only peer-reviewed content. Non-English content was also excluded because most recognized academic contributions are usually published in English.

**Figure 2.**
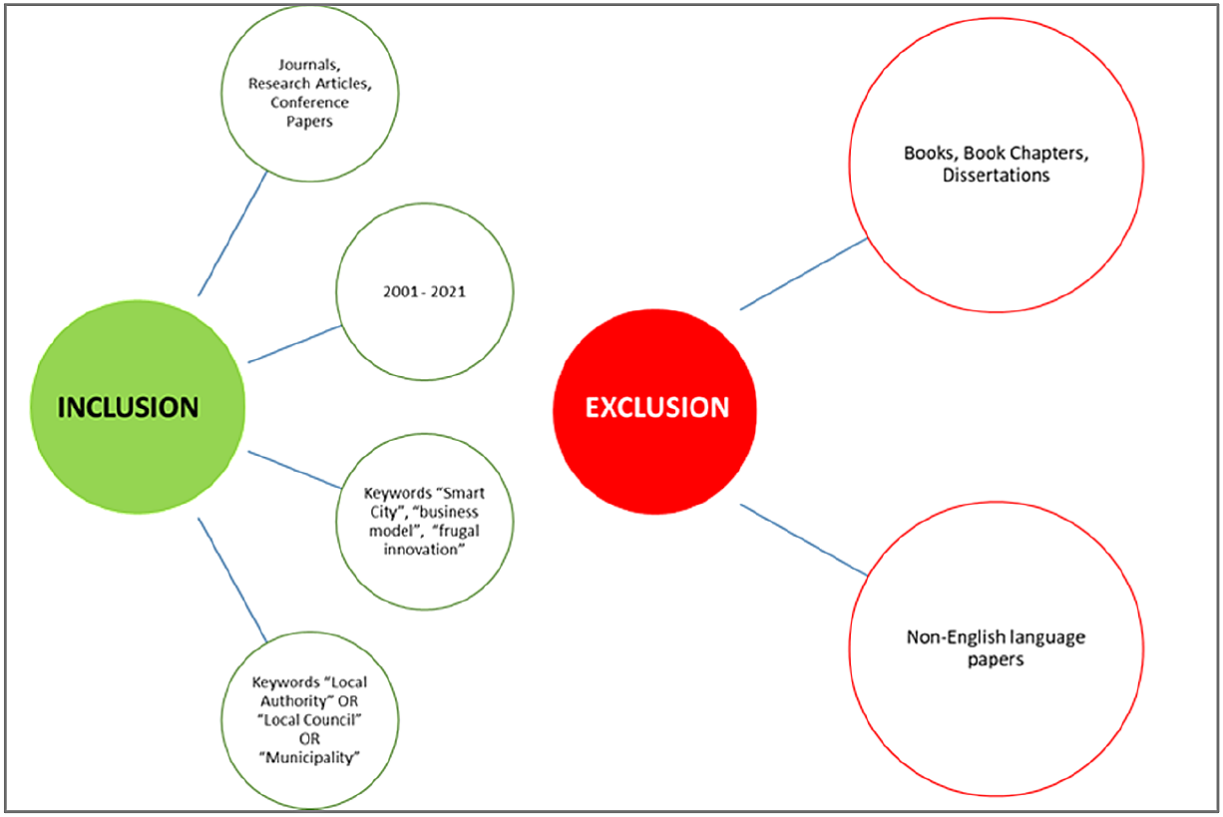
Inclusion and exclusion for the review.

Figure 2 summarizes the analysis’ inclusion/exclusion criteria.

*Keywords*

This study focuses on business models for local authorities to implement smart city initiatives and the applicability of the frugal innovation theory. Thus, the keywords used were as follows:

(1) ‘Smart city’ AND ‘business model’ AND ‘frugal innovation’ and
(2) ‘Local authorities’ OR ‘local councils’ OR ‘municipalities’, which many authors use interchangeably.

Other keywords, such as ‘performance’, ‘inclusivity’, ‘technology’ and ‘success factor’ were also introduced to paint a comparative landscape on the breadth of literature available covering smart cities.

*Search strategy*

The main strategy adopted was to comb through the vast online database to discover literature that discussed smart city business models for local authorities. The covered online databases were Emerald, ProQuest, Scopus, IEEE Xplore, ScienceDirect, and Springer Link. The identified keywords are then looked up in all the databases above to indicate the number of literature available related to the specific topics.

### Stage 3: extracting and synthesizing data

The findings of the above exercise are indicated in Table 1. ^8^

**Table 1.** Search results based on pre-identified keywords.

Keywords combinations									
No.	Online Database	Smart City	Smart City AND Performance	Smart City AND Sustainability	Smart City AND Success Factors	Smart City AND Technology	Smart City AND Business Model	Smart City AND Business Model AND Local Authority OR Local Council OR Municipal	Smart City AND Business Model AND Local Authority OR Local Council OR Municipal AND Frugal Innovation
1	Emerald	159	17	20	1	58	3	-	-
2	ProQuest	350	279	134	1	313	19	6	-
3	Scopus	26,807	4,846	1,377	51	9,569	212	6	-
4	IEEE	3,458	574	137	-	1,350	10	-	-
5	Science Direct	1,889	447	467	3	803	27	1	-
6	Springer	610	326	89	6	449	29	4	-
	**Total:**	**33,273**	**6,489**	**2,224**	**62**	**12,542**	**300**	**17**	**-**

**Figure 3.**
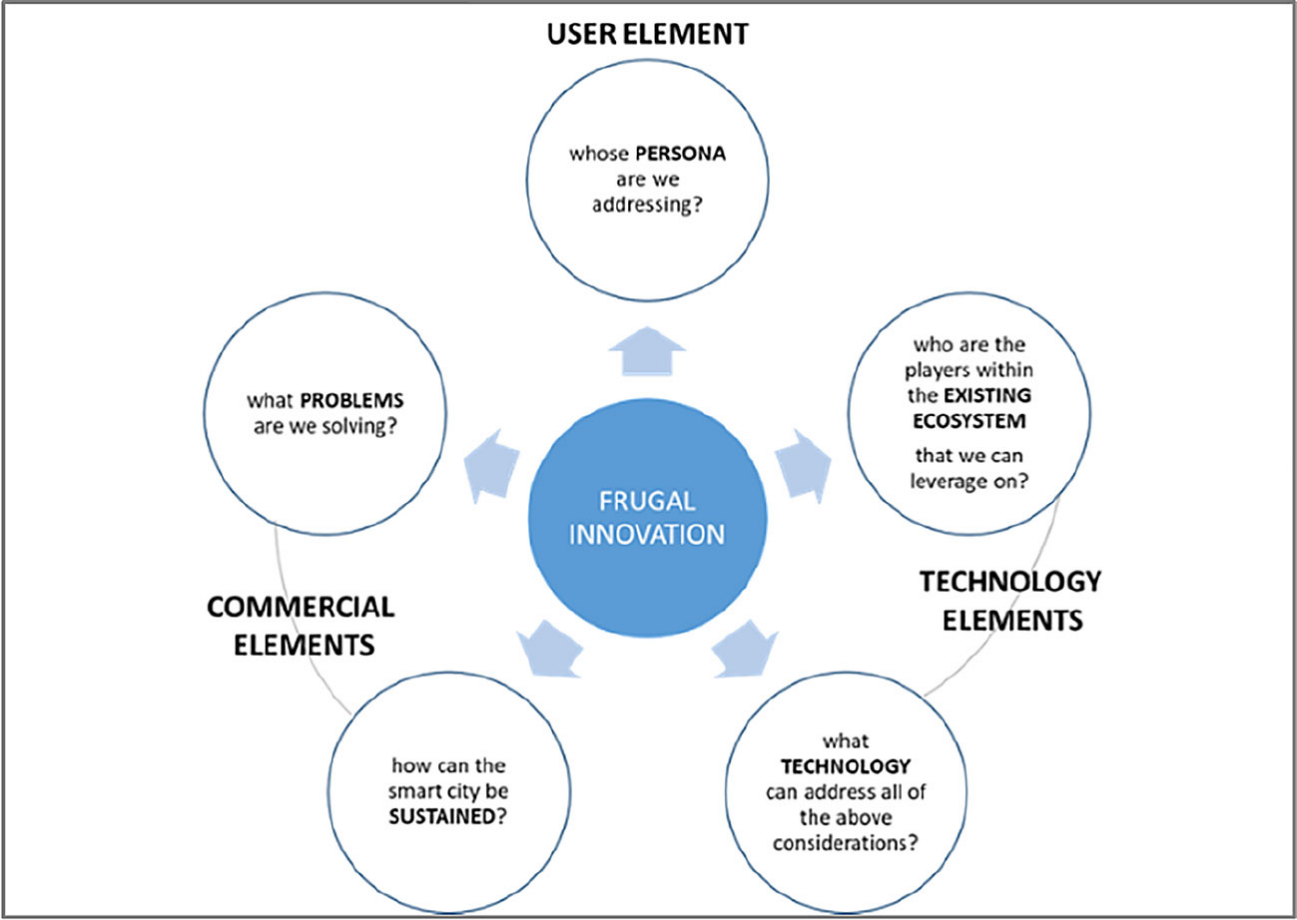
Conceptual framework on the applicability of frugal innovation in smart city business model.

From the above exercise, the narrowed search results related to the smart city business model for local authorities were reviewed to exclude any further irrelevant research. The PRISMA methodology was adopted to facilitate further the inclusion and exclusion process illustrated in Figure 3. Nevertheless, the Tranfield method remains the primary methodology adopted for this systematic literature review. Stages four and five of the Tranfield method will be discussed in the following sections.

## Results

### Summary of core papers

With more than 33,273 papers, the topic of smart cities is well-covered. Specific topics within smart cities are also well-covered, including smart city technology, with 12,542 papers representing 38% from the total smart city research, smart city performance (6,489/19%) and smart city sustainability (2,224/7%). However, only 300 papers on smart city business models were found, representing only 0.9% of the research. When further analysed, a further gap regarding smart city business models for local authorities was observed, with only 17 papers found, out of which 13 were excluded because the discussions were not specifically related to business models. Further investigation revealed no papers linking the smart city business model in local authorities to frugal innovation theory. The summary of the papers reviewed are presented in Table 2.

**Table 2.** Summary of papers reviewed.

	Authors (Year)	Findings	Business model elements	Business model mentioned	Frugal innovation
1.	^9^McLean and Roggema (2019)	Innovative policy and regulation are needed to transform consumers to prosumers, thereby achieving greater financial benefit, ownership and control	• Next-Gen infrastructure approach	Prosumer	Not mentioned
			• Innovative regulation		
2.	^10^Papageorgiou et al. (2020)	The presented SPN business model framework could serve as a common platform for communication purposes and open innovation	• End users	• Outright purchase	Not mentioned
			• API gateways	• Subscription	
			• System integrators	• Freemium with limited feature	
			• External service providers	• Free ad-supported model	
			• City/municipalities	• Free city-sponsored model.	
3.	^11^Anthopoulos et al. (2016)	In practice, cities adopt different business models than the ones suggested in literature. The ownership business model is the most optimal, the Open Business Model the most preferred and the Municipal-Owned-Development as an alternative model		• Ownership	Not mentioned
				• Open	
				• Municipal-owned-development	
4.	^12^Liu et al. (2021)	PPPs focus more on building new or improving existing infrastructure		• Public-private partnership (PPP)	Not mentioned
5.	^13^Schrotter and Hürzeler (2020)	EXCLUDED. Paper discusses technology perspective			
6.	^14^Spruytte et al. (2019)	EXCLUDED. While the paper discusses the revenue/cost model of WiFi deployment, the focus of the study is not on the business model, but rather the technical angle on how to deploy the models			
7.	^15^Liu et al. (2020)	EXCLUDED. The paper discusses technical aspects of IOT deployment			
8.	^16^Ramos et al. (2021)	EXCLUDED. The paper discusses technical aspects of national database related to health			
9.	^**17**^Kumar et al. (2021)	EXCLUDED: The paper discusses flood mitigation policies			
10.	^**18**^Ng (2018)	EXCLUDED. The paper discusses unconventional business models in the context of regulatory sandboxing policies, but not the business model itself			
11.	^**19**^Cohen (2018)	EXCLUDED. The paper discusses business model in the context of ride sharing, and its relationship with the local government			
12.	^**20**^Ji et al. (2014)	EXCLUDED. The paper discusses design and implementation of IOT in smart cities from a technical perspective			
13.	^**21**^Spiliotopoulou and Roseland (2020)	EXCLUDED: The paper discusses sustainable community development from a social perspective			
14.	^**22**^Ford et al. (2021)	EXCLUDED. The paper mentions new business model should be incorporated into the smart local energy system, but does not discuss the model itself			
15.	^**23**^Gallico (2020)	EXCLUDED. The paper focuses on standard of sustainability and multiculturalism specifically for fashion industry			
16.	^**24**^McShane and Grechyn (2019)	EXCLUDED. The paper discusses business model as one of the ingredients for the deployment of local government WiFi			
17.	^**25**^Octavianthy and Purwanto (2018)	EXCLUDED. While the paper extensively discusses many types of business models, it is in relation to a very specific scenario, i.e. the implementation of smart energy in Depok, Indonesia, with limited discussion on the theory behind the business models		• Fiscal incentive	
				• Low loan interest	
				• Viability gap fund	
				• Increased tipping fee	
				• Grant	
				• Electricity pricing	

### Research gap one: research on smart city business

Table 2 mentions several business models. However, discussion on the business models’ theory to allow a generic application to local authority smart city initiatives, has been insufficient.

### Research gap two: frugal innovation application in smart city business model

The exercise in Table 2 revealed that no papers linked the smart city business model in local authorities to frugal innovation theory, thus presenting a potential research gap.

## Discussion

From the many engagements with the Malaysian local authorities relating to smart cities, five common ingredients of a smart city regularly emerge: the people, the needs, financial sustainability, inclusivity and technology. Technology plays a key role in enabling a smart city, but it is important not to fall into technological determinism; whereby a smart city’s function is blindly dictated by technology. ^26^ Any smart city is about the citizens’ needs. A smart city merely uses technology to address those specific needs. Thus, a smart city should intuitively adapt and respond to the needs of its citizens.

A good business model would increase a smart city’s chances of success. Firstly, a good business model would ensure financial sustainability for the city’s smart services and reduce the chances of those services being abandoned. Secondly, a good business model would also include existing players from within the city’s ecosystem. By having these elements, there would be less resistance, a lower entry barrier and a greater sense of ownership, encouraging the services’ longevity. With this in mind, the findings of this study are as follows:

1. Table 1 reveals that the topic of the smart city is well-covered by research and specific sub-topics within smart cities, including technology, performance, and sustainability. However, a research gap is found in the smart city business model for local authorities, with only 17 papers found, out of which only four were somewhat relevant. Thus, the study posits that gaps exist between the problems faced in the industry itself and available research on the subject, and that the business models that are researched are still insufficient to address the problems faced by the local authorities.
2. Table 2 shows that the following business models were mentioned concerning local authorities’ smart city initiatives:
  • PPP
  • Prosumer
  • Outright purchase
  • Subscription
  • Freemium
  • Ad-supported
  • City-sponsored
  • Ownership
  • Open model
  • Municipal-owned development
  However, the papers did not extensively cover the elements that are needed for a smart city business model. Thus, it would be challenging to apply the business models mentioned to a generic local authority smart city initiative.
3. Based on the findings, there are no papers related to frugal innovation theory. However, literature and evidence in frugal innovation prove that it can be a game-changer for a sustainable business model. ^27^ The framework applying the concept of frugal innovation to the elements of smart city mentioned above is submitted to depict frugal innovation applicability in the smart city business model. In this model, it is submitted that frugal innovation can be applied to the three elements that make up a successful smart city business model: the user element, the commercial elements and the technological elements. When considering each of these three, the question that must consistently be asked is how they all can be successfully achieved with the least resources, as depicted in Figure 3 below.

A limitation of this study is the number of keywords selected. Keyword selections are based on research focus. However, there is possibility of obtaining more articles if the keywords are expanded to field of study that are not specific in nature such as municipality. This could possibly be publication bias.

## Conclusions

Based on our findings, future research can study frugal innovation initiatives in smart cities. Future research can also study alternative smart city business models for local authorities. Finally, future research could also assess the impact of frugal innovation on smart cities operations efficiency. This can potentially address how government agencies can convince private corporations to participate in smart city initiatives by convincing them that capital investment made into smart city initiatives are financially beneficial.

Financial sustainability is crucial for implementing smart cities, and a good business model would support this sustainability. Unfortunately, there are not a lot of studies dedicated to this topic. Frugal innovation has been applied to many other areas and industries; no literature supports the notion that it has been applied to the smart city business model. Further research on this concept would be beneficial because frugal innovation theory is a proven model for success, ^6^ and thus would potentially benefit an initiative that ultimately aims to provide a better life for all.

## Data availability

### Underlying data

Figshare: Summary of papers reviewed for business models for local authority smart city https://doi.org/10.6084/m9.figshare.14877123. ^8^

This project contains the following underlying data:

• Data file 1. (summary of papers, xlsx format).

### Reporting guidelines

Figshare: PRISMA checklist and flow diagram https://doi.org/10.6084/m9.figshare.16722991

Data are available under the terms of the Creative Commons Attribution 4.0 International (CC BY 4.0)
