# Supplementary material for: Sustainable business model for local council’s smart city initiatives: a systematic literature review
Source: F1000Res. 2022 Jun 22;10:1066. Originally published 2021 Oct 20. [Version 2] doi: 10.12688/f1000research.73373.2 (PMC12749557; doi:10.12688/f1000research.73373.2)
Supplement: Peer review history

# Reviewer report: Reviewer response for version 2

**DOI:** 10.5256/f1000research.135369.r435068

**Published:** 2025-12-29

**Authors:** Xiaodong Yang

**Affiliations:** 1 Shihezi University, Shihezi, Xinjiang, China

Thanks to the editor for the invitation, some improvement suggestions are as follows:

 The core connotation and adaptation logic of frugal innovation have not been clearly explained, resulting in a lack of convincing "why it applies to local government smart city business models". The paper only mentions "doing more with less resources" and does not distinguish its essential differences from traditional low-cost models and lean models, nor does it explain its unique advantages in the public sector scenario (such as adapting to fiscal constraints and emphasizing people's livelihood orientation).

 The paper uses "performance" and "inclusiveness" as auxiliary keywords, but does not include the core concern "financial sustainability" into the core search combination, and does not consider synonymous substitutions (for example,"local government","municipal government" and "city council" need to be fully covered, and "cost-effective innovation" can be added as a synonymous expression of frugality and innovation). It is recommended to reconstruct the keyword combination with "(smart city) AND (business model OR financial sustainability) AND (local authority OR municipality OR local government) AND (frugal innovation OR cost-effective innovation)" as the core, and at the same time eliminate auxiliary keywords that are weak related to the research topic to avoid interference from irrelevant literature and improve screening accuracy.

 From 33,273 initial documents to 300 "Smart City Business Model" documents to 17 "Local Government-Related" documents, the exclusion criteria at each step only mentioned "irrelevant" in general and did not clearly state the specific basis for "irrelevant"., and the specific reasons for the exclusion of the 13 documents were not marked one by one. It is recommended to supplement the complete PRISMA flow chart, detailing the number of screening and exclusion criteria at each step (such as inconsistent topics, non-local governments, and no business model discussion), and explain the selection basis for the four included core documents to ensure that the study is repeatable.

 The paper only lists 10 business models such as PPP and subscription, and does not analyze the applicable conditions of each model in the smart city scenario (such as what types of smart city projects the PPP model is suitable for and what prerequisites are required), financial sustainability performance (such as investment return cycle, risk sharing mechanism), nor does it discuss obstacles to the implementation of the model in conjunction with the administrative attributes and fiscal constraints of local governments. It is recommended to increase comparative analysis of business models, build an evaluation framework from four dimensions: "capital needs, operation difficulty, public welfare adaptability, and financial sustainability", and combine cases in existing literature (such as the effectiveness of subscription system for smart parking in a city, Failure lessons from a regional PPP model) to clarify the advantages and disadvantages of different models and application scenarios.

 The three-element framework of "user-business-technology" proposed in the paper only stays at the conceptual level and does not explain the specific implementation path of frugal innovation under each element (for example,"minimum resources" in the technical elements refer to simplifying the technical architecture and reusing existing facilities, or adopting open source technology), nor does it define the interaction between various elements. It is recommended to refine the dimensions of the framework, for example, add the sub-dimensions of "revenue source, cost control, risk sharing" to the "commercial elements", and clarify specific directions such as "technology selection, facility reuse, and operation and maintenance simplification" in the "technical elements", and combine 1-2 virtual cases (such as frugal and innovative design of local government smart government services) to demonstrate the application logic of the framework.

 The paper focuses on Malaysia's 150 local councils, but does not analyze their unique fiscal structure (such as tax dependence, transfer payment ratio), the financing difficulties of existing smart city projects (such as whether any projects have stalled due to funding shortages), and policy environment constraints (such as regulatory barriers to public-private partnerships), resulting in a lack of realistic adaptability of research conclusions.

 The paper does not discuss why there is a gap in research on the relationship between frugal innovation and smart city business models. Is it due to lack of academic understanding, lack of practical cases, or is it limited in research methods? Nor has it learned from the application experience of frugal innovation in other public service fields (such as education and medical care) to provide ideas for filling the gap.

Are the rationale for, and objectives of, the Systematic Review clearly stated?

Partly

Is the statistical analysis and its interpretation appropriate?

Partly

If this is a Living Systematic Review, is the ‘living’ method appropriate and is the search schedule clearly defined and justified? (‘Living Systematic Review’ or a variation of this term should be included in the title.)

Not applicable

Are sufficient details of the methods and analysis provided to allow replication by others?

Partly

Are the conclusions drawn adequately supported by the results presented in the review?

Partly

Reviewer Expertise:

green innovation

I confirm that I have read this submission and believe that I have an appropriate level of expertise to confirm that it is of an acceptable scientific standard.

# Reviewer report: Reviewer response for version 2

**DOI:** 10.5256/f1000research.135369.r432997

**Published:** 2025-12-11

**Authors:** Nurul Huda Ibrahim

**Affiliations:** 1 nhuda_i@uum.edu.my, Sintok, Malaysia

**Strengthen the Theoretical Justification for Frugal Innovation**

 While the article identifies a literature gap, it does **not sufficiently explain**:

 What frugal innovation *is* beyond “doing more with less”.

- Why it is relevant to smart cities.
- How principles from engineering/industry translate to urban governance.
- Whether frugal innovation has been applied in **any** local government functions.

This lack of conceptual grounding makes the research aim feel disjointed.

 Several ambiguities need correction:

 Describe whether keyword searches targeted title/abstract/keywords/full text.

 Explain why 300 smart city business model papers became only 17—include explicit inclusion/exclusion criteria.

- Define what is meant by “quality studies.”
- Demonstrate how subjective bias in screening was minimized.

The current discussion is descriptive. The analysis should include:

- Interpretation of why business model research is scarce.
- The relationship between model types (PPP, prosumer, freemium, municipal-owned, etc.) and local government contexts.
- Why existing models may not suit Malaysian local councils.
- How the ecosystem (stakeholders, governance structure, financial constraints) influences model suitability.

The paper currently lacks:

- Contributions to theory (e.g., how frugal innovation expands smart city business-model literature).
- Contributions to practice (how local authorities can apply findings).
- Policy implications (for Malaysian PBTs).
- Suggestions for future empirical research designs.

Improve Abstract

- Condense background sentences.
- Clarify purpose and findings more concisely.
- Remove redundant phrases such as “The data is tabulated…”

Provide clearer definitions in the introduction.

 Include:

- A standard definition of “smart city” (e.g., Giffinger et al.).
- Distinction between “smart city,” “digital city,” and “sustainable city”.

Reconsider Table 2 Positioning

 Explain in text how it leads to the research gaps.

 Overall Recommendation

**Recommendation: *Major Revision Required***

 The article has a strong foundation and clearly identifies an important gap in smart city research. However, several methodological and theoretical weaknesses must be addressed before it can be considered scientifically robust.

Are the rationale for, and objectives of, the Systematic Review clearly stated?

Partly

Is the statistical analysis and its interpretation appropriate?

Not applicable

If this is a Living Systematic Review, is the ‘living’ method appropriate and is the search schedule clearly defined and justified? (‘Living Systematic Review’ or a variation of this term should be included in the title.)

Not applicable

Are sufficient details of the methods and analysis provided to allow replication by others?

Partly

Are the conclusions drawn adequately supported by the results presented in the review?

Yes

Reviewer Expertise:

pedagogy, communication

I confirm that I have read this submission and believe that I have an appropriate level of expertise to confirm that it is of an acceptable scientific standard, however I have significant reservations, as outlined above.

# Reviewer report: Reviewer response for version 2

**DOI:** 10.5256/f1000research.135369.r194068

**Published:** 2023-08-24

**Authors:** Robert Cowley

**Affiliations:** 1 Department of Geography, King's College London, London, England, UK

Drawing on a systematic search of the extensive peer-reviewed literature, the article demonstrates that the applicability of a ‘frugal innovation’ business model has not attracted academic attention in relation to the smart city. By extension, it encourages researchers to explore this topic, in order to inform more sustainable modes of funding smart city programmes.

 The basic finding about the gap in the literature is fairly straightforward, but clearly evidenced. For the article to be published, however, a rather more substantial framing of the research aims is required, along with a more generative set of conclusions.

 A key reservation for me, as a reader not previously familiar with the idea of frugal innovation, is that I am left feeling unpersuaded about the reasons why this particular business model is worthy of future attention. I am sure there are all manner of other topics – and business models - which are not covered in the smart city literature – but that absence does not in itself justify further research. Certainly, if it can be shown that funding models are currently inadequate, then there would be good reason to explore new approaches, and especially those which have proven successful in somehow related governance contexts. As things stand, the authors do mention that frugal innovation has been applied to engineering and industrial design, but do not explain why it would therefore be “worth investigating” in relation to the smart city. If the smart city was largely a question of engineering or industrial design, then the relevance of frugal innovation would perhaps be self-evident. But can private sector business models be applied so easily to local government contexts? The article claims that “literature and evidence in frugal innovation prove that it can be a game-changer for a sustainable business model” (p.10), but in what ways in particular? Why specifically might this be relevant to local authorities? 

 Further explaining the need for research into this topic (beyond the existence of a research gap) seems vital to me if the article is to be indexed. A starting point would be to provide further insight into what frugal innovation involves – beyond the buzzwords flagged up in Figure 3. Please tell us more about how this differs from other approaches, and what others might have said about its potential applications. Are there any other fields of local authority activity, for example, where it has been applied? Which of its basic principles seem clearly relevant to smart city development? In what ways specifically does it depart from a ‘conventional business model’? (Is there only one conventional/traditional business model in any case?) Is it perhaps a new idea which has spread rapidly across lots of different fields? 

 Ideally, I would also like to see some concrete evidence that current funding models are not sustainable. I don’t have reason to doubt this, and the fact that it came up as a significant issue at the 2018 conference mentioned on p.3 is a good start. ‘Smart city’ programmes usually consist of specific projects in reality (which may in reality be rather small-scale) – but would it be possible to provide examples of envisioned projects in Malaysia which will probably not happen due to funding obstacles? Any examples from elsewhere of grand visions being cut down to scale, or specific initiatives being dropped for financial reasons?

 For the article to be published, I would also like to see the conclusions fleshed out a little. Drawing on an earlier discussion of the reasons why frugal innovation might be relevant (as proposed above), what particular research questions should researchers be asking? What might the limitations of frugal innovation be? What more theoretical interventions might be enabled by such research? What kinds of methods do you have in mind? 

 I have a few more specific comments for the authors to address:

 Abstract: the three sentences from “Local authorities” to “smart city initiatives” could easily be condensed into one.

 P3: “Traditionally, cities are funded by government, for example, through tax collection”. This point could be much sharper. Surely, city services etc. are typically provided through tax collection – but what do you mean by “cities”? Isn’t much of the city funded by private investment? And what do you mean by ‘traditionally’? For decades, a wide array of governance and funding models have been at play in all sorts of cities, involving the private sector and all sorts of other stakeholders.

 The three objectives simply repeat the three research questions. I’m not sure in any case that the third objective could be met from the present study (and it hasn’t been). I suggest rephrasing the third one as more of an overall research aim - for example: "Overall, the article aims to encourage researchers to explore the potential relevance and limitations of the frugal innovation business model to the funding of smart city projects in local authority areas”.

 Methods: the second and third paragraphs of the “Ethics” subsection are not related to ethics. Wouldn’t it be better to place the ethics point at the end of the methods section (or have it as a footnote)?

 Methods: clarify key point: were you searching on keywords appearing in the front matter of the articles, or looking for their appearance in the main text of the articles? (I’m guessing the latter.)

 Methods: the definition of ‘quality’ is a little odd. Are all published journal articles of good quality? If peer review is the criterion, are all conference papers properly peer reviewed?  “Journal/conference papers and research articles” is fuzzy – are research articles not papers? I think it would be better simply to specify that the evidence base consisted of articles and conference papers listed by the online databases shown on page 5.

 Figure 2 doesn’t add value - it just repeats what has appeared in the text. Couldn’t this be deleted?

 Page 7: “When further analysed…” It would be good to reword the end of this sentence for clarity – is the point that they contain no substantial discussion of business models, even though these words appear in the text?

 Page 7 “A good business model would increase a smart city’s chance of success”. This does not seem like a useful analytical point. Isn’t it self-evident?

 Page 8 (point 2): this just lists out what appears in Table 2. Surely, what is needed here is discussion and analysis of some sort.

 Finally, I haven’t gone through closely looking for typos, but I happened to notice the following, in case it’s helpful:

 Abstract: “prosperous living conditions initiatives” could be more elegantly worded. Maybe “quality of life initiatives”?

 Abstract: “data is” should really be “data are”.

 Abstract: “The data is tabulated for clear expression of knowledge gaps” – not sure what you mean. Could you just delete this? (Does it just mean that some findings have been presented as tables?)

 Abstract: delete comma after “further” in the final sentence.

 Abstract and first para: put single speech marks around ‘frugal innovation’ the first time it appears, since it is a technical term.

 Intro second para: “the paper concludes” (not concluded).

 Smart City Phenomenon: delete comma after “world”.  “Tipped” should also be rephrased – maybe just “exceeded”.

 Smart City Phenomenon: “in regards that” – rephrase eg “with regards to the need for…”

 P4: “systematic” rather than “systemic”.

 P7: delete comma after “initiatives”.

Are the rationale for, and objectives of, the Systematic Review clearly stated?

Partly

Is the statistical analysis and its interpretation appropriate?

Not applicable

Are sufficient details of the methods and analysis provided to allow replication by others?

Partly

Are the conclusions drawn adequately supported by the results presented in the review?

Yes

Reviewer Expertise:

Sustainable cities; smart cities; urban governance

I confirm that I have read this submission and believe that I have an appropriate level of expertise to state that I do not consider it to be of an acceptable scientific standard, for reasons outlined above.

# Reviewer report: Reviewer response for version 2

**DOI:** 10.5256/f1000research.135369.r141737

**Published:** 2022-08-16

**Authors:** Marco Tregua

**Affiliations:** 1 Department of Economics, Management, Institutions, University of Naples Federico II, Naples, Italy

Dear Authors, 

 I have still concerns about this manuscript. 

 Please consider the recommendations below:

 The need to address (or refer to) smart cities from a practical perspective has not led to any change. Please, either justify the missing change or change the content.

 The structure of your work is not yet described at the end of the introduction. 

 I would reinforce research questions through recalling recent calls for papers/contributions. 

 In one of the research questions you recall frugal innovation, but the literature review doesn't offer a clear description of what it is and how it affects a smart city. 

 Method:

 You wrote that you only reviewed 'quality studies'. Did you mean qualitative or there is another way you selected publications according to the quality of their content?

 The framework comes out of blue; moreover, it is unclear how you moved from 300 contributions to just 17. Criteria for selection should be carefully described, even more if one considers you started with a dataset of 33k+ publications. 

 The title of the research gap one is probably incomplete, since you wrote 'business' instead of 'business model'.

 Discussion:

 There are two severe issues, since (a) once again the business models of top companies around the world are not cited; and (b) the discussion starts with reference to Malaysia and there is no way to track back the use of the 17 publications you selected. Was the framework used? Moreover, some considerations are very basic, e.g. 'a good business model would also include existing players from within the city’s ecosystem': I would doubt the opposite, since stakeholders/players are mentioned in every single paper on smart city and also in well reputed models.

 Finally, I am wonder why there are no implications from this manuscript, nor theoretical neither empirical, apart from a consideration on frugal innovation on p. 10.

 There are typos and minor mistakes, please proofread your manuscript (e.g., 'An early paper describes a smart city and one that is independent': I think you meant 'as' instead of 'an')

 Kind regards

Are the rationale for, and objectives of, the Systematic Review clearly stated?

Partly

Is the statistical analysis and its interpretation appropriate?

Not applicable

Are sufficient details of the methods and analysis provided to allow replication by others?

Yes

Are the conclusions drawn adequately supported by the results presented in the review?

No

Reviewer Expertise:

Innovation management, international management

I confirm that I have read this submission and believe that I have an appropriate level of expertise to confirm that it is of an acceptable scientific standard, however I have significant reservations, as outlined above.

# Reviewer report: Reviewer response for version 1

**DOI:** 10.5256/f1000research.77019.r120222

**Published:** 2022-02-09

**Authors:** Marco Tregua

**Affiliations:** 1 Department of Economics, Management, Institutions, University of Naples Federico II, Naples, Italy

Dear Authors,

 There are some interesting proposals in your manuscript as well as some relevant shortcomings, please take a look at my comments below and - first and foremost - take care of proposing implications.

**Introduction**:

 In the very first lines you refer to smart (and sustainable) city initiatives but you recall digital city services, please be aware of the difference and note it it at the beginning. I would suggest you to see what scholars mention about the comparison of these topics. You can refer to several publications, I can mention few as examples: Hollands, R. G (2008) ^1^, Tregua, M., D’Auria, A., & Bifulco, F.

 (2014) ^2^, and Ishida, T. (2017). ^3^

 You recalled the notion of smart city, I'd do that with a definition and a quote from one of the most important works in this domain, e.g., Giffinger, R. (2015), ^4^ or Fernandez-Anez, V., Fernández-Güell, J. M., & Giffinger, R. (2018). ^5^

 Finally, I agree with your justification of the reason to perform this study, but since you deal with business models and several big firms have proposed their solutions for smart cities, I would wrap up the introduction by recalling a model or two proposed by big companies such as IBM, Microsoft, Cisco, and so on.

 Please complete the introduction with a description of the structure of the chapter.

**Method**

 This section is well written and reliable; anyway, there are two missing elements:

 a) I would start from previous reviews, if any, to justify the reason for your study.

 b) I would describe how you avoided subjectivity in your selection of extant literature.

**Results**:

 Referring to table 2 is not enough to justify/describe your two research gaps. Please debate it some more. Table 2 simply reports the evidence you picked up, while it is needed to clearly state why what you reported led you to highlight a research gap.

 Moreover, I wonder why this section is called results, since there are no 'real' results. This section is not actually hosting results, but

 it is a description of the process you carried out.

**Discussion**:

 I agree with your consideration on smart cities, as per the text in the first 5 lines. This consideration would have worked even better with a definition of smart city in the first section of this manuscript.

*Sub-section 2 of the Discussion*:

 This view is limited, since big companies have their business model for smart cities. Thus, either you should explicitly consider them too, or highlight if these models are recalled by scholars in the contributions you analysed. Considering big companies would lead you to observe how business models for smart cities were set. Moreover, local agencies very often involve big companies in running smart cities projects and they cooperate in defining a business model suitable for a specific area. This is also relevant in considering 'how' and 'why' a big company gets involved in such projects.

**Conclusions**:

 I am very surprised by the lack of implications. I agree with some of your considerations, but such a manuscript would have a very limited usefulness with nor theoretical neither practical implications and appears as an opinion paper instead of being a research paper.

Are the rationale for, and objectives of, the Systematic Review clearly stated?

Partly

Is the statistical analysis and its interpretation appropriate?

Not applicable

Are sufficient details of the methods and analysis provided to allow replication by others?

Yes

Are the conclusions drawn adequately supported by the results presented in the review?

No

Reviewer Expertise:

Innovation management, international management

I confirm that I have read this submission and believe that I have an appropriate level of expertise to confirm that it is of an acceptable scientific standard, however I have significant reservations, as outlined above.

## References

[1] : Will the real smart city please stand up?. *City* .2008;12(3) : 10.1080/13604810802479126 303-320 10.1080/13604810802479126

[2] : Digital City towards Smart City: a theoretical overview. *Proceedings 2nd International Virtual Scientific Conference* .2014;

[3] : Digital City, Smart City and Beyond. *Companion: Proceedings of the 26th International Conference on World Wide Web Companion* .2017;1151-1152 Reference source

[4] : Smart City Concepts: Chances and Risks of Energy Efficient Urban Development.579: 10.1007/978-3-319-27753-0_1 3-16 10.1007/978-3-319-27753-0_1

[5] : Smart City implementation and discourses: An integrated conceptual model. The case of Vienna. *Cities* .2018;78: 10.1016/j.cities.2017.12.004 4-16 10.1016/j.cities.2017.12.004
